# Reliability and construct validity of the modified Finnish version of the 9-item patient health questionnaire and its associations within the biopsychosocial framework among female health-care workers with sub-acute or recurrent low back pain

**DOI:** 10.1186/s12891-020-03832-y

**Published:** 2021-01-07

**Authors:** J. H. Suni, T. Virkkunen, P. Husu, K. Tokola, J. Parkkari, M. Kankaanpää

**Affiliations:** 1 UKK Institute Health Promotion Research, Kaupinpuistonkatu 1, 33500 Tampere, Finland; 2 Pirkanmaa Hospital District, Physical and Rehabilitation Medicine Outpatient Clinic, Teiskontie 35, 33520 Tampere, Finland

## Abstract

**Background:**

Health-care workers have an increased risk for chronic low back pain (LBP) leading to reduced workability. Depression, a highly prevalent, costly and disabling condition, is commonly seen in patients with sub-acute LBP. This study investigated the psychometric properties and construct-validity of a modified 9-item Patient Health Questionnaire (PHQ-9-mFIN) in female health-care workers with sub-acute LBP.

**Methods:**

Reliability (internal consistency, test-retest repeatability) was assessed using standard methods. Construct validity of the PHQ-9-mFIN was assessed as level of depressive symptoms (PHQ-9-mFIN: 0–4 none, 5–9 mild, ≥10 at least moderate) against the RAND 36 Health Survey, a valid measure of health-related quality of life (HRQoL). Additionally, the strength of the association between the levels of PHQ-9-mFIN and selected biopsychosocial factors was determined.

**Results:**

The internal consistency of the PHQ-9-mFIN was high (Cronbach’s α = 0.82) and the test-retest repeatability scores (*n* = 64) were moderate: Pearson’s correlation was 0.73 and Intraclass Correlation Coefficient (ICC) 0.73 (95% CI: 0.58 to 0.82). Construct validity (Spearman correlation) against the Physical and Mental component items and their summary scales of the RAND 36 were much higher for the Mental (range, − 0.40 to − 0.67 and − 0.64) than for the Physical (range, − 0.08 to − 0.43 and − 0.22). There was a clear stepwise association (*p* < 0.001) between the levels of depressive symptoms and General health (physical component, range, 59.1 to 78.8). The associations with all items of the Mental components were strong and graded (*p* < 0.001). All participants had low scores for Bodily pain, regardless of the level of depressive symptoms. There was a strong association (*p* ≤ 0.003) between the levels of PHQ-9-mFIN and multisite pain, lumbar exertion and recovery after workdays, neuromuscular fitness in modified push-ups, workability, and fear of pain related to work.

**Conclusions:**

The PHQ-9-mFIN showed adequate reliability and excellent construct validity among female health-care workers with recurrent LBP and physically strenuous work.

**Trial registration:**

NCT01465698.

**Supplementary Information:**

The online version contains supplementary material available at 10.1186/s12891-020-03832-y.

## Background

Low back pain (LBP) affects people of all ages. Today, LBP is one of the leading causes of disability and contributes to the huge global disease burden, with the highest prevalence being in working-age populations [1–3]. Moreover, between 1990 and 2015, there was a 54% increase in disability-adjusted life-years [2]. In most people, LBP is described as non-specific, as it is not always possible to identify a specific nociceptive cause [3]. At the individual level, musculoskeletal pain reduces health-related quality of life (HRQoL) both physically and mentally [4]. Across all member states of the European Union, LBP and other musculoskeletal disorders are the leading cause of work disability, sickness absence from work, and loss of productivity [5].

Depression is a highly prevalent, costly, and disabling condition [6] that is commonly seen in patients with subacute LBP [7–9]. In 2018, LBP was the leading worldwide cause of years lived with disability, whereas depressive disorders were ranked third [6]. Currently, it is unknown whether depression is a cause of LBP. However, cross-sectional data among patients with subacute LBP indicate that men and women with LBP have a significantly higher depressive symptoms score compared with those with no pain [7]. Prospective findings on the course of acute and subacute LBP suggest that depressive symptoms may have an adverse effect on the prognosis of LBP [8]. Individuals with depressive symptoms may therefore have an increased risk for developing an episode of LBP in the future, with a higher risk in those patients with more severe levels of depressive symptoms [9].

Health care is one of the employment sectors that has significantly higher rates of sickness absence from work with a subsequent negative impact on employee health, health-care delivery, and patient health [10]. Indeed, the annual prevalence of LBP among hospital nurses and nurses’ aides in Europe is between 51 and 57%, and new high-risk groups include home and long-term care nurses and physiotherapists [11]. According to a Scottish Health Board database (comprising approximately 12,000 health-care employees), musculoskeletal disorders (MSDs) accounted for 24% and mental health problems 20% of the total number of working days lost over a 6-year period [10]. Of all sickness absence events, LBP had the highest incidence at 34%. Interestingly, the highest burden of work loss due to both musculoskeletal and mental conditions was observed among nurses and midwives [10]. In Finland, MSDs account for a third of the overall costs of sickness absence and a fifth of the costs of all disability pensions [12].

The Patient Health Questionnaire-9 (PHQ-9) is commonly used as a screening instrument for depressive symptoms in primary care. The PHQ-9 is brief, self-administered, easy to score, and well validated for detecting and monitoring changes in depression severity [13, 14]. There is a Finnish version of the original PHQ-9 questionnaire, which has been targeted for clinical use to define more severe depressive symptoms [15]. Our group has produced a modified Finnish version of the PHQ-9 questionnaire in terms of a shorter verbal design, and we have replaced questions 6, 7, 8, and 9. By making these modifications, we aimed to produce a depressive symptom scale that would be valid in detecting mild subjective depressive symptoms, and therefore be more applicable for the healthy Finnish working population.

When studying the validity of new or modified measurement properties, reliability and validity issues must be checked according to the consensus-based standards for the selection of health measurement instruments (COSMIN) [https://www.cosmin.nl/]. COSMIN provides criteria for the measurement properties of patient-reported outcome measures (PROMs). The criteria include reliability (internal consistency, repeatability, and measurement error), validity (content, criterion and construct validity), and responsiveness (measurement property responsiveness). The COSMIN checklists also provide a tool to evaluate the methodological quality of studies on measurement properties [16].

The aim of the present study was to investigate the reliability (internal consistency, test-retest repeatability) and construct validity of the modified Finnish version of PHQ-9 (PHQ-9-mFIN) as well as its associations within the biopsychosocial framework [17, 18] among female health-care workers with recurrent non-specific LBP and physically strenuous work [19–22].

The hypothesis was that PHQ-9mFIN is a valid measurement property to assess depressive symptoms among female health-care workers with recurrent non-specific LBP who are still able to work. It was expected that PHQ-9mFIN would have a strong negative association with the mental part of HRQoL [13].

## Methods

### Data collection, study design, and sample

This study contains cross-sectional baseline data from the NURSE-RCT (NCT01465698) [19–22] and data from a small test-retest repeatability study (*n* = 64) among volunteer participants of the NURSE-RCT. The inclusion criteria of the RCT were women aged 30 to 55 years, had worked at their current job for at least 12 months, and intensity of LBP was at least 2 on the Numeric Rating Scale (scale 0–10) during the past 4 weeks. The exclusion criteria were prior serious back injury (fracture, surgery, disc protrusion); chronic LBP defined by a physician or self-report of continuous LBP for 7 months or more; disease or symptoms that limit participation in moderate intensity neuromuscular exercise; regular engagement in neuromuscular-type exercise more than once a week; pregnant or recently delivered. In total, 439 women responded to the screening questionnaire. Of these, 56% (*n* = 245) met the inclusion criteria and 11% (*n* = 26) refused to participate in the baseline measurements. The main back-related reasons for exclusion were intensity of LBP of less than 2 on the Numeric Rating Scale (22%) and having had continuous LBP for more than 7 months (12%) [19].

**Fig. 1:**
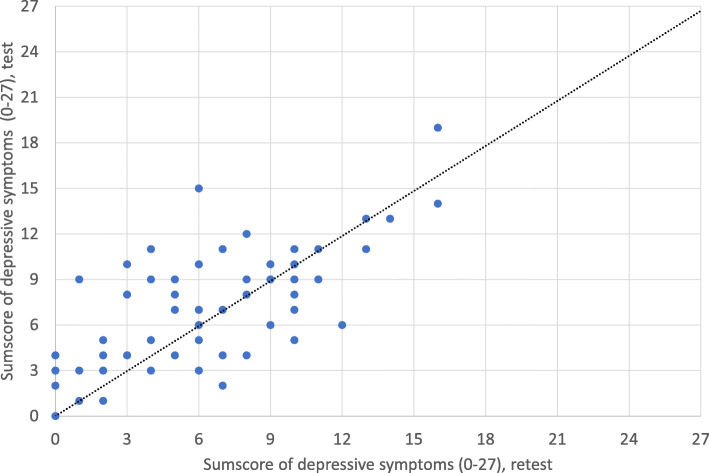
The scatter plot presenting the one week test-retest results of the modified Finnish version of the 9-items Patient Health Questionnaire

The test-retest data on selected questionnaire items, including PHQ-9-mFIN, were collected from sub-studies 2 and 3 of the NURCE-RCT in the fall of 2014 as part of the participants 24-month (sub-study 2) and 12-month (sub-study 3) follow-up measurements performed at the UKK Institute, Tampere, Finland (see Fig. 1 and Table 1 of the study protocol) [19]. The participants first filled out the standard NURSE-study questionnaire [19] at home (1st measurement) 1 week before attending the follow-up measurements conducted at the UKK Institute. The participants then filled out a repeatability questionnaire (2nd measurement) during the follow-up measurement session at the UKK institute. All participants provided their written informed consent to participate in the study to a research secretary at the beginning of the baseline measurements. The study protocol of NURSE-RCT is available at the following address: https://www.ncbi.nlm.nih.gov/pmc/articles/PMC5117067/pdf/bmjsem-2015-000098.pdf [19]. The Regional Ethics Committee of the Expert Responsibility area of Tampere University Hospital (ETL code R08157) approved the study protocol.

**Table 1:** Descriptive results on the questions of the modified Finnish PHQ-9 among female healthcare workers with recurrent non-specific low back pain (*N* = 219)

Over the past week, how often have you been bothered by any of the following problems (1–9)	Item scores:^a^ 0%	1%	2%	3%	Missing (n, %)
1. *Lack of enthusiasm for doing anything*	20.2	55.5	18.3	6.0	(1, 0.5)
Original: Little interest or pleasure in doing things					
2. *Feeling depressed*	30.7	51.8	14.7	2.8	(1, 0.5)
Original: Feeling down, depressed, or hopeless					
3. *Have trouble getting to sleep or staying asleep*	64.2	31.7	2.3	1.8	(1, 0.5)
Original: Trouble falling or staying a sleep, or sleeping too much					
4. *Feeling low in energy or slowed down*	48.6	41.3	8.3	1.8	(1, 0.5)
Original: Feeling tired or having little energy					
5. *Have a poor appetite*	44.0	45.9	8.7	1.4	(1, 0.5)
Original: Poor appetite or overeating					
6. *Cry easily or feel like crying*	30.7	56.0	11.0	2.3	(1, 0.5)
Original: Feeling bad about yourself —or that you are a failure or have let yourself or your family down					
7. *Feeling bored or having little interest in doing things*	21.2	50.2	22.6	6.0	(2, 0.9)
Original: Trouble concentrating on things, such as reading the newspaper or watching television					
8. *Feeling yourself lonely*	22.9	34.4	30.7	11.9	(1, 0.5)
Original: Moving or speaking so slowly that other people could have noticed? Or the opposite					
9. *Feeling hopeless about the future*	63.8	31.2	4.1	0.9	(1, 0.5)
Original: Thoughts that you would be better off dead of or hurting yourself in some way					

^a^0 = *hardly ever*/not at all; 1 = *seldom*/several days; 2 = *often*/more than half of the days; 3 = *very often*/nearly every day

### Assessment methods

#### Assessment of depressive symptoms

The nine questions of the PHQ-9-mFIN and the original PHQ-9 [13] are provided in Table 1. In the modified questionnaire, participants were asked to report, how often they had been bothered by any of the following symptoms over the past week: 1) lack of enthusiasm for doing anything, 2) feeling depressed, 3) have trouble getting to sleep or staying asleep, 4) feeling low in energy or slowed down, 5) poor appetite, 6) cry easily or feel like crying, 7) feeling bored or having little interest in doing things, 8) feeling lonely, and 9) feeling hopeless about the future.

In the original PHQ-9, [13] the symptoms were assessed over a period of 2 weeks. The response options of the modified PHQ-9-mFIN were 0 = hardly ever, 1 = seldom, 2 = often, and 3 = very often. The wording of the scoring (0–3) of both versions is provided at the bottom of Table 1. The responses for the nine questions were summarized as PHQ-9-mFIN total score (0–27), which was then categorized into three groups of depressive symptoms: scores 0–4 as None, 5–9 as Mild, and ≥ 10 as at least Moderate.

#### Assessment of health-related quality of life

HRQoL was assessed using the RAND-36 item health survey 1.0 that includes eight separate scales: 1. Physical functioning, 2. Role Functioning/Physical, 3. Bodily Pain, 4. General Health, 5. Vitality, 6. Social functioning, 7. Role functioning/Emotional, and 8. Mental Health.

First, we studied the correlations of the total score of the PHQ-9-mFIN against the four Physical (1–4) and Mental components (5–8) (0–100) of the RAND-36 and their corresponding summary scores (0–100), which are presented in Table 2. Second, we studied the associations between the eight components and the two summary scores of the RAND-36 with the level of depressive symptoms according to the PHQ-9-mFIN [23].

**Table 2:** Intercorrelations between the modified Finnish version of the nine item Patient Health Questionnaire (PHQ-9-mFIN) and the Physical and Mental component and summary scales of health-related quality of life (RAND 36 Health Survey)

Components of RAND 36 Health Survey	PHQ-9-mFIN^a^	*p*-value
Physical functioning	−0.25	< 0.001
Role functioning/physical	−0.08	0.273
Bodily Pain	−0.08	0.251
General health	−0.43	< 0.001
***Physical component summary scale***	−0.22	0.002
Vitality	−0.62	< 0.001
Social functioning	−0.44	< 0.001
Role functioning/emotional	−0.40	< 0.001
Mental Health	−0.67	< 0.001
***Mental component summary scale***	−0.64	< 0.001

^a^*N* = 210, missing = 9 (4.1%)

#### Assessment of biopsychosocial factors

The association of the PHQ-9-mFIN was assessed within the biopsychosocial model (i.e., Pain, Functioning, Participation, Individual factors) which provides a useful framework for understanding the factors that may contribute to chronicity in LBP and are important targets for interventions among patients with subacute or recurrent LBP [17, 18]. Standard methods were used to assess the background variables and selected biopsychosocial factors of the NURSE-RCT at baseline: intensity of LBP in the Visual Analog Scale (VAS) [24], number of musculoskeletal pain sites [25], lumbar exertion after workdays, [26] and recovery after work [27]. Additionally, we measured the muscular fitness of the trunk and upper-body using the modified push-ups test [28, 29]. Work ability was assessed with work ability score [30] and work stress as effort-reward imbalance [31]. The Fear-Avoidance Beliefs Questionnaire [32] was used to measure beliefs regarding fear and avoidance towards work and physical activity.

We present the descriptive data of the study population based on the level of depressive symptoms, assessed by the PHQ-9-mFIN, and using the original categories [13] described above. The aim was to acquire knowledge of those factors that are related to increasing levels of depressive symptoms among female health-care workers with recurrent LBP and physically strenuous work [19–22].

### Statistical methods

Descriptive data are presented as percentages for categorical variables and mean values with standard deviation or 95% confidence intervals (CI) for continuous variables. The internal consistency of the items of the PHQ-9-mFIN was assessed by Cronbach’s α coefficient. A commonly used rule for describing internal consistency when using Cronbach’s alpha is: α ≥ 0.9 = excellent, α ≥ 0.8 = good, ≥ 0.7 = acceptable, α ≥ 0.6 = questionable, α ≥ 0.5 = poor, α < 0.5  = unacceptable [33]. The 1-week test-retest repeatability of the total score (0–27) of the PHQ-9-mFIN was assessed by Pearson correlation and Intraclass Correlation Coefficient (ICC). ICC was calculated using a 2-way mixed effects model and assuming absolute agreement. The test-retest scores in the form of a scatter plot are presented in Fig. 1.

The construct validity of the PHQ-9-mFIN was assessed against the RAND 36, a validated Finnish questionnaire [23], with Spearman correlation (non-normally distributed data) and one-way analysis of variance (ANOVA) using Sidak-adjusted *p*-values for multiple comparisons between groups (normally distributed data). Associations between the categorized PHQ-9-mFIN and biopsychosocial factors were tested with Kruskall-Wallis H due to non-normal distributions. In this study, the internal consistency and construct validity were defined and tested according to the COSMIN checklist Box A and Box F [16]. All statistical analyses were conducted by KT using SPSS statistics software, version 25 (IBM, Chicago, IL).

## Results

The mean age of the participants was 46 years, mean time in their current job was 11 years, and 70% worked shifts [20]. The majority of the participants were nurses (45%) or nurses’ aides (41%). Of the participants, 28% were current smokers; 59% had a body mass index (BMI) of 25 or more indicating overweight, and 18% were obese (i.e., BMI ≥30) [34].

The majority (65%) of the participants reported a pain in the back duration [25] of less than 3 months, 40% had clinically meaningful intensity of LBP (i.e., ≥40 mm in VAS) [24], and 12% experienced daily pain [25]. Almost a third (31%) of the participants reported musculoskeletal pain in three or more body sites of at least moderate intensity (≥4 in the numeric rating scale 0–10) [25]. The majority (78%) of the female health-care workers reported no days of sickness absence due to LBP during the preceding 6 months [22].

### Descriptive results of the PHQ-9-mFIN

Of the nine questions in the PHQ-9-mFIN (see Table 1) questionnaire, “Feeling yourself lonely” (question 8) had the highest proportion of scores of 2 and 3 indicating a higher level of depressive symptoms (20.7 and 11.9%, respectively), followed by “Feeling bored or having little interest in doing things” (question 7; 22.6 and 6.0%), and “Lack of enthusiasm for doing anything” (question 1; 18.3 and 6.0%). The highest proportion of zero scores (no depressive symptoms) was detected for the questions “Have trouble getting to sleep or staying asleep” (question 3; 64.2%) and “Feeling hopeless about the future” (question 9; 63.8%). The mean value of the PHQ-9-mFIN in the present study population was 7.4 (range, 0 to 27).

### Reliability and construct validity

The internal consistency of the PHQ-9-mFIN, assessed by Cronbach’s α, was 0.82. The Pearson’s test-retest repeatability correlation (*n* = 64) over the 1-week test-retest interval was 0.73 and ICC was 0.73 (95% CI: 0.58 to 0.82). The scatter plot (Fig. 1) indicates that the repeatability is lowest between the scores from 3 to 7 and highest from 9 up to the highest possible (i.e., 27).

The correlations (Spearman) of the Physical and Mental component items and their summary scales of the RAND 36-Item Health Survey [23] with PHQ-9-mFIN were much higher for the Mental components (range, − 0.40 to − 0.67) and their summary scale (− 0.64) when compared to those of the Physical components (range, − 0.08 to − 0.43, summary − 0.22), see Table 2.

Of the Physical components (see Table 3), Bodily pain had the lowest mean score of 63.0. However, the differences between the levels of depressive symptoms (PHQ-9-mFIN: None 0–4, Mild 5–9, at least Moderate ≥10) were small (range, 61.3 to 64.5) and statistically non-significant. Conversely, there was a clear stepwise association (*p* < 0.001) between the levels of depressive symptoms and General health (range 59.1 to 78.8), i.e., those with a Moderate level in PHQ-9-mFIN had the poorest health. Physical functioning had the highest mean score of 85.5, indicating good physical functioning in the present study population. However, the differences between the three levels of depressive symptoms were statistically significant (*p* < 0.001). The Physical summary score (mean 72.9) showed a small (range, 67.8 to 77.6) graded association (*p* = 0.002) between the levels of depressive symptoms. The mean Physical summary score in the None-symptoms group was lower than that of the mean Mental summary score (77.6 vs. 87.5).

**Table 3:** Associations between the depressive symptoms, measured with the modified Finnish version of the nine item Patient Health Questionnaire (PHQ-9-mFIN), with the Physical and Mental components and their summary scores (0–100) of health-related quality of life (RAND-36 Health Survey)

PHQ-9-mFIN^a^	Physical functioning	Role funct. Physical	Bodily pain	General health	Physical summary	Role funct. Emotional	Vitality	Mental Health	Social functioning	Mental summary
N = 210^b^	Mean (sd)	Mean (sd)	Mean (sd)	Mean (sd)	Mean (sd)	Mean (sd)	Mean (sd)	Mean (sd)	Mean (sd)	Mean (sd)
**Total:**	85.5 (13.5)	74.0 (32.5)	63.0 (19.0)	69.0 (16.5)	72.9 (15.4)	83.3 (28.5)	62.9 (18.8)	76.9 (14.6)	83.6 (19.1)	76.6 (16.8)
**By level**^b^**:** ANOVA										
0–4 (*n* = 60)	90.3 (9.9)^3^	76.9 (29.6)	64.5 (18.2)	78.8 (14.0)^2,3^	77.6 (13.2)^3^	94.4 (13.7)^3^	75.4 (13.7)^2,3^	87.7 (7.0)^2,3^	92.3 (11.3)^3^	87.5 (9.0)^2,3^
5–9 (*n* = 92)	85.7 (12.5)^3^	74.2 (32.6)	63.1 (18.6)	68.8 (14.6)^1,3^	73.0 (14.5)	85.5 (25.3)^3^	65.1 (14.3)^1,3^	77.9 (10.4)^1,3^	86.4 (15.9)3	78.7 (11.6)^1,3^
≥ 10 (*n* = 58)	80.1 (16.2)^1,2^	70.7 (35.4)	61.3 (20.5)	59.1 (16.0)^1,2^	67.8 (17.6)^1^	68.4 (35.6)^1,2^	46.3 (17.8)^1,2^	64.1 (16.2)^1,2^	70.3 (22.9)^1,2^	62.3 (19.9)^1,2^
*p*-value:	< 0.001	0.58	0.68	< 0.001	0.002	< 0.001	< 0.001	< 0.001	< 0.001	< 0.001

^a^categories of depressive symptoms: 0–4 as None; 5–9 as Mild; ≥10 as at least Moderate

^b^missing = 9 (4.1%)

^1^denotes statistically significant (*p* < 0.05, sidak-adjusted) difference between category and none (0–4) of depressive symptoms

^2^denotes statistically significant (*p* < 0.05, sidak-adjusted) difference between category and mild (5–9) of depressive symptoms

^3^denotes statistically significant (*p* < 0.05, sidak-adjusted) difference between category and at least moderate (≥10) of depressive symptoms

Regarding the Mental components, there was a strong graded and statistically significant (*p* < 0.001) association between the levels of depressive symptoms and each component item and the mean summary score. The lowest score of all component items, including Physical components, was found for Vitality among those with a Moderate level of depressive symptoms (46.3). The group without depressive symptoms had the two highest mean scores of all for the component items “Role functioning/ Emotional” and “Social functioning,” with scores of 94 and 92, respectively. The mean Mental summary score of those with Moderate depressive symptoms was slightly lower than that of the mean Physical summary score (62.3 vs. 67.8).

### Associations between PHQ-9-mFIN and biopsychosocial factors

Descriptive results of the study population within the biopsychosocial framework based on the level of depressive symptoms measured with the PHQ-9-mFIN are presented in Table 4. The proportion of female health-care workers with at least moderate symptoms (score ≥ 10) was 28% (*n* = 61) as was the percentage of those with no depressive symptoms (scores 0–4; n = 61).

**Table 4:** Descriptive data of female healthcare workers with recurrent low-back pain (LBP) by the level of symptoms of depression measured with the modified Finnish version of the Patient Health Questionnaire-9 (PHQ-9-mFIN)

Depression categories of PHQ-9-mFIN:	None 0–4 (*n* = 61)	Mild 5–9 (*n* = 96)	Moderate ≥ 10 (*n* = 61)	All (*N* = 218)	Kruskal-Wallis H	Missing (%)
PHQ-9-mFIN: mean (95% confidence interval, CI)	2.5 (1.1 to 5.2)	7.0 (4.3 to 9.6)	13.1 (5.6 to 20,7)	7.4 (− 1.6 to 16.5)		1 (0.5)
**Pain**	mean (95% CI)	mean (95% CI)	mean (95% CI)	mean (95% CI)	*p*-value	
Intensity of LBP: VAS (0–100 mm)	30.2 (25.0 to 35.4)	37.3 (32.5 to 42.2)	40.0 (34.4 to 45.6)	36.1 (33.1 to 39.1)	0.039	2 (0.9)
Number of musculoskeletal painsites (0–4)	2.8 (2.5 to 3.0)	3.3 (3.0 to 3.6)	3.6 (3.2 to 4.0)	3.2 (3.1 to 3.4)	0.001	2 (0.9)
Lumbar exertion after workdays(1–5)	2.7 (2.5 to 3.0)	3.1 (3.0 to 3.3)	3.3 (3.0 to 3.6)	3.1 (2.9 to 3.2)	0.003	4 (1.8)
**Functioning**						
Recovery after workdays (1–5)	2.2 (2.0 to 2.4)	2.6 (2.4 to 2.8)	3.2 (3.0 to 3.4)	2.7 (2.5 to 2.8)	< 0.001	1 (0.5)
Modified push-ups (n/40s)	9.9 (9.2 to 10.7)	9.1 (8.5 to 9.7)	8.0 (7.2 to 8.8)	9.0 (8.6 to 9.5)	0.001	7 (3.2)
Predicted VO_2_max (ml/min/kg)^a^	32.9 (31.8 to 34.0)	31.8 (30.8 to 32.8)	30.3 (29.0 to 31.6)	31.7 (31.0 to 32.4)	0.009	8 (3.7)
**Participation/Disability**						
Work Ability Score (0–10)	8.4 (8.1 to 8.6)	7.9 (7.7 to 8.1)	7.0 (6.6 to 7.4)	7.8 (7.6 to 8.0)	< 0.001	1 (0.5)
Work stress: effort-rewardimbalance (0.2–5):	1.5 (1.4 to 1.6)	1.6 (1.5 to 1.8)	1.7 (1.6 to 1.8)	1.6 (1.6 to 1.7)	0.014	2 (0.9)
**Individual factors**						
FABs^b^ related to work (0–48) ^c^	11.8 (10.2 to 13.4)	13.9 (12.6 to 15.1)	14.1 (12.5 to 15.8)	13.4 (12.5 to 14.2)	< 0.001	2 (0.9)
FABs related to physicalactivity (0–30)	8.0 (6.2 to 9.9)	11.4 (9.9 to 12.9)	13.2 (10.8 to 15.6)	10.9 (9.9 to 12.0)	0.099	10 (4.6)

^a^6 min’ walk test; ^b^*FABs* Fear Avoidance Beliefs; ^c^questions 10, 15 and 16 were excluded as non-relevant in the present study population

The mean intensity of LBP during the past 4 weeks measured with VAS was at a clinically meaningful level of 40 mm [24] among those with moderate depressive symptoms and at the lowest level (i.e., 30 mm) among those with no symptoms (*p* = 0.039). There were stepwise associations (*p* ≤ 0.003) between the level of depressive symptoms and the number of musculoskeletal pain sites [25], lumbar exertion after workdays [26], recovery after work days during the past 4 weeks [27], neuromuscular fitness in modified push-ups test [28, 29], Work Ability Score [30], and fear of pain [32] related to work, but not that related to physical activity. The effort-reward imbalance (0.2–5), an indicator of work stress [31], slightly increased with the level of depressive symptoms (*p* = 0.014).

## Discussion

The nine item Patient Health Questionnaire is a screening tool used worldwide for major depressive disorder in different health-care settings with acceptable diagnostic properties at a cut-off score of 10 or above [35, 36]. The score of 10 was recently shown to maximize combined sensitivity and specificity overall and for subgroups [36]. The validity of both the PHQ-9 [37] and the Mental Component Summary score of the Short Form-36 Health Survey [38] to screen major depressive symptoms has been established in patients with chronic LBP.

The present study investigated the psychometric properties of a modified Finnish version of the PHQ-9 among female health-care workers with sub-acute or recurrent LBP. We are unaware of any previous validation studies of the PHQ-9 with this target group. The RAND 36-Item Health Survey provides benefits as a general functional health status measure and a criterion measure to study the construct validity of the PHQ-9-mFIN [38]. The assessment of the relationships of a variety of biopsychosocial factors with the level of depressive symptoms, measured with the PHQ-9-mFIN, provides knowledge of the possible risk factors for long-term LBP among those with or without depressive symptoms.

### Psychometric properties

Cronbach’s α of 0.82 indicates that the internal consistency of the PHQ-9-mFIN is good and in line with the results of previous studies using the original PHQ-9 in primary care patients in Latvia [39] (Latvian version α = 0.79; Russian version α = 0.81) and Thailand (α = 0.79) [40] as well as among the general population in China (α = 0.86) [41] and Hong Kong (α = 0.82) [42].

The correlation coefficient of 0.73 indicates acceptable repeatability for the 1-week test-retest interval. Three earlier studies with a 2-week test-retest interval reported similar (0.76) [40, 42] and higher (0.86) [41] correlations. The scatter plot presented in Fig. 1 further shows that the repeatability is higher when the depressive symptom score is at least 9 (i.e., close to the moderate level of ≥10) or when the score is very low, from 0 to 3, indicating no depressive symptoms.

The original PHQ-9 assesses symptoms during the past 2 weeks. We chose to use the 1-week time-frame, as it was the time duration during which the participants wore accelerometers for “objective” assessment of physical activity [43] and sedentary behavior [44]. Furthermore, “Subjective” questionnaire data on physical activity and/or exercise are also usually collected for a 1-week period. Because physical activity and exercise are recommended treatments for moderate depression [45] as well as for recurrent LBP [46], we chose to collect data on both for a period of 1 week.

As expected, the correlations of the Physical component subscales (range, ─0.08 to ─0.43) and their mean Summary score (─0.22) of the RAND-36 with PHQ-9-mFIN were much lower than those of the Mental component subscales (range, ─0.40 to ─0.67, Summary ─0.64). When compared to previous studies among the general population [41, 47], the correlations of the Mental scores with PHQ-9-mFIN in the present study were somewhat higher, indicating a strong association between the two.

The results on the associations between the Physical and Mental component scores of the RAND-36 with the level of depressive symptoms according to PHQ-9-mFIN, using the original cut-off points for None (0─4), Mild (5─9), and Moderate (≥10) depression [13], indicated reduced HRQoL (i.e., scores < 70 out of 0─100) in the RAND-36 component items Bodily Pain, regardless of the level of depressive symptoms and General health, Vitality, and Mental health among those with at least a moderate level of symptoms. Thus, the present study group of female health-care workers with subacute or recurrent LBP who engaged in strenuous physical work for the back suffered from Pain (mean 63.0) regardless of whether they had depressive symptoms or not. Those with at least moderate symptoms lacked Vitality (i.e., were tired, mean 46.3), and their General health (mean 59.1) and Mental health (mean 64.1) were reduced when compared with optimal levels.

### Association with the biopsychosocial factors

The main interest for assessing the associations was to find possible biopsychosocial risk factors for adverse future events among the female health-care workers engaged in strenuous physical work and experiencing recurrent LBP with or without depressive symptoms. Among patients with recurrent LBP, depressive symptoms are expected to have an adverse effect on the prognosis [8].

In our previous cross-sectional study among the present study population, work-related Fear-Avoidance Beliefs (*p* < 0.001), lumbar exertion (*p* = 0.003), depressive symptoms (*p* = 0.01), and recovery after work (*p* = 0.03) best explained work ability [21]. Multi-site musculoskeletal pain has also been associated with poor physical work ability among health-care workers. Indeed, the magnitude of the association is likely to increase with a higher number of pain sites [48]. In Finland, co-occurrence of musculoskeletal pain and depressive symptoms is strongly related to poor self-rated physical work ability [49].

A clear dose-response relationship has been reported between increasing levels of depressive symptoms and the risk for long-term sickness absence (LTSA) [50]. Furthermore, the adverse effect of non-clinical depressive symptoms manifested at relatively low scores [50]. In Finland, musculoskeletal pain, but not depression, is associated with thoughts of early retirement [49]. Among Danish health-care workers, depressive symptoms and the number of musculoskeletal pain locations were associated with an increased risk of LTSA in those individuals who did not have comorbid symptoms [51].

## Conclusion

The modified Finnish version of the PHQ-9 is shorter in overall verbal design and it has replaced the psychologically most devastating statements of questions 6, 7, 8, and 9 with more positive ones to be more applicable in interventions among apparently healthy worker populations or in large scale population studies. The PHQ-9-mFIN showed adequate reliability and excellent construct validity among the study group of female health-care workers with recurrent LBP and physically strenuous work for the lower back.

## Supplementary Information

**Additional file 1.** The PHQ-9-mFIN questions.

## Data Availability

The datasets used and analyzed during the current study are available from Dr. Jaana Suni (JS; jaana.suni@ukkinstituutti.fi) of the NURSE RCT study upon reasonable request.

## Abbreviations

BMI: Body mass index
HRQoL: Health-related quality of life
LBP: Low back pain
LTSA: Long-term sickness absence
PHQ-9: 9-item Patient Health Questionnaire
PHQ-9-mFIN: Modified Finnish version of the 9-item Patient Health Questionnaire
RAND-36: RAND 36-Item Health Survey
VAS: Visual analogue scale
