# Supplementary material for: Reliability and construct validity of the modified Finnish version of the 9-item patient health questionnaire and its associations within the biopsychosocial framework among female health-care workers with sub-acute or recurrent low back pain
Source: BMC Musculoskelet Disord. 2021 Jan 7;22:37. doi: 10.1186/s12891-020-03832-y (PMC7792227; doi:10.1186/s12891-020-03832-y)
Supplement: Supplementary file 1 — Additional file 1. The PHQ-9-mFIN questions. [file 12891_2020_3832_MOESM1_ESM.pdf]

Supplementary file. The PHQ-9-mFIN questions.

Over the past week, how often have you been bothered by any of the following problems

1. Lack of enthusiasm for doing anything?
3 = very often
2 = often
1 = seldom
0 = hardly ever

2. Feeling depressed?
3 = very often
2 = often
1 = seldom
0 = hardly ever

3. Have trouble getting to sleep or staying asleep?
3 = very often
2 = often
1 = seldom
0 = hardly ever

4. Feeling low in energy or slowed down?
3 = very often
2 = often
1 = seldom
0 = hardly ever

5. Have a poor appetite?
3 = very often
2 = often
1 = seldom
0 = hardly ever

6. Cry easily or feel like crying?
3 = very often
2 = often
1 = seldom
0 = hardly ever

7. Feeling bored or having little interest in doing things?
3 = very often
2 = often
1 = seldom
0 = hardly ever

8. Feeling yourself lonely?
3 = very often
2 = often
1 = seldom
0 = hardly ever

9. Feeling hopeless about the future?
3 = very often
2 = often
1 = seldom
0 = hardly ever
